# Prevalence of Opioid Use and Associated Healthcare Outcomes in Rome IV Irritable Bowel Syndrome in the United Kingdom

**DOI:** 10.1111/apt.70400

**Published:** 2025-10-08

**Authors:** Mohsin F. Butt, Vivek C. Goodoory, Cho Ee Ng, Christopher J. Black, Alexander C. Ford, Maura Corsetti, Peter Paine

**Affiliations:** ^1^ NIHR Nottingham Biomedical Research Centre, Nottingham University Hospitals NHS Trust and the University of Nottingham Nottingham UK; ^2^ Nottingham Digestive Diseases Centre, Translational Medical Sciences, School of Medicine, University of Nottingham Nottingham UK; ^3^ Leeds Institute of Medical Research at St. James's, University of Leeds Leeds UK; ^4^ Leeds Gastroenterology Institute, St. James's University Hospital Leeds UK; ^5^ County Durham and Darlington NHS Foundation Trust Durham UK; ^6^ Manchester Academic Health Sciences Centre UK; ^7^ Gastroenterology, Salford Royal Foundation Trust Salford UK

**Keywords:** DGBI, IBS, opioid

## Abstract

**Background:**

The United Kingdom (UK) has one of the highest worldwide opioid prescription rates per capita. International guidelines caution against the use of opioids for chronic non‐malignant pain, including irritable bowel syndrome (IBS).

**Aims:**

To study the prevalence and impact of regular opioid use among people with Rome IV IBS in community settings in the UK.

**Methods:**

We collected demographic, quality of life (QoL), psychological symptoms, and healthcare utilisation data from 752 people with Rome IV IBS. Participants were recruited via electronic mailshot in July 2021 through ContactME‐IBS, a UK registry of persons diagnosed with IBS primarily by a general practitioner or gastroenterologist.

**Results:**

Among participants, 148 (19.7%) reported taking an opioid regularly. The prevalence of opioid use did not vary across IBS subtypes (*p* = 0.10). Disease‐specific and generic QoL were lower among opioid users than non‐users (*p* < 0.001, both analyses). Compared with non‐users, a greater proportion of opioid users had anxiety or depression (*p* < 0.001, both analyses) and greater overall activity impairment (*p* < 0.001). The direct healthcare costs related to investigations (*p* = 0.003), IBS‐related medications (*p* < 0.001), and unplanned hospital attendances (*p* < 0.001) were greater among participants who used opioids. Opioid users also expressed a greater willingness to accept the risk of death in return for a cure of IBS symptoms from a hypothetical medication (*p* = 0.002).

**Conclusion:**

Approximately one in five people with Rome IV IBS used opioids on a regular basis. Compared with non‐users, opioid users had worse QoL, greater psychological impairment, and higher direct healthcare costs.

## Graphical abstract

One in five people with Rome IV irritable bowel syndrome use opioids on a regular basis. Compared with non‐users, opioid users had worse quality of life, greater psychological impairment, and higher direct healthcare costs.

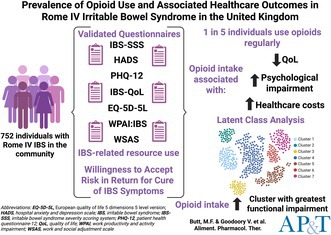

## 1 Introduction

The United Kingdom (UK) has the world's highest opioid prescription rates per capita [1]. The gastrointestinal burden of opioid intake among people in the UK was reflected in the Rome Foundation Global Epidemiology study, which demonstrated a higher rate of Rome IV opioid‐induced constipation in the UK than in Canada and the United States of America (USA) [2, 3]. Aside from constipation, other common gastrointestinal side effects of opioids include nausea, vomiting, and bloating [4, 5]. Visceral pain, which features prominently in numerous disorders of gut‐brain interaction (DGBI), including irritable bowel syndrome (IBS), may also be amplified by opioid‐induced central sensitisation [6].

IBS, which affects 4% of the global population [2, 7], is a DGBI defined by the Rome criteria as altered bowel movements (either constipation, diarrhoea, or both) in the presence of abdominal pain related to defaecation [8]. However, the conventional Rome bowel‐habit classification system has been challenged by latent class analytical approaches that integrate both gastrointestinal and psychological symptoms, yielding seven reproducible IBS patient clusters that range from those with predominantly gastrointestinal symptoms to those with high psychological burden [9, 10, 11, 12].

International guidelines caution against the use of opioids in the management of chronic non‐malignant pain [13, 14], and the iatrogenic harm that may arise from their use in the management of IBS is addressed in UK national guidelines [15, 16]. Previous studies suggest that up to one in four patients with IBS consume opioids and highlight an association between opioid use and poorer quality of life (QoL), greater symptom severity, and higher healthcare resource utilisation [17, 18]. However, these observations were based on tertiary care patient populations [17, 18], data from outside the UK, specifically France [17], and have focused on IBS with constipation without using validated questionnaires [18].

To our knowledge, the prevalence of opioid use across various subtypes of Rome IV IBS, along with its effects on gastrointestinal and psychological symptoms, QoL, work and activity impairment, and healthcare utilisation, has not been assessed among individuals in the UK. The primary aims of this cross‐sectional study were to evaluate the prevalence of regular opioid use among individuals with Rome IV IBS in the community and the relationship between opioid use and healthcare outcomes. The secondary aim of the study was to determine how opioid consumption varied between the seven previously defined latent clusters [9, 10, 11, 12], to provide novel insights into prescribing patterns and help identify subgroups at greatest potential risk of opioid‐related harm.

## 2 Methods

### 2.1 Participants and Setting

We recruited individuals registered with ContactME‐IBS, a national UK registry, coordinated by County Durham and Darlington NHS Foundation Trust, consisting of 4280 members with IBS who have expressed an interest in volunteering for research. Data analyses using this registry have been reported elsewhere [10, 19, 20, 21, 22, 23, 24, 25]. Individuals self‐identify to the registry as having IBS and enrol online by completing a short questionnaire about their bowel symptoms. Among all registrants, at the time of enrolment, 2268 (53%) had consulted a general practitioner (GP), 1455 (34%) a gastroenterologist, and 557 (13%) had not seen a doctor for IBS. There were no exclusion criteria apart from the inability to understand written English.

We contacted all individuals registered with ContactME‐IBS, via electronic mailshot, in July 2021. Those willing to participate in the study completed an online questionnaire and non‐responders received a reminder email in August 2021. Those who participated in the study were given the opportunity to win one of three gift cards (worth £200, £100, or £50) in return for completing the questionnaires. The University of Leeds research ethics committee approved the study in March 2021 (MREC 20‐051).

### 2.2 Data Collection and Synthesis

#### 2.2.1 Demographic and Symptom Data

We collected basic demographic data, including age, gender, lifestyle (tobacco and alcohol consumption), ethnicity, marital status, educational level, and annual income. We also asked respondents to state whether their IBS symptoms commenced after an acute enteric infection. We defined the presence of IBS according to Rome IV criteria [26], assigning the presence or absence of Rome IV IBS according to the scoring algorithm proposed for their use [8]. We categorized each IBS subtype according to the criteria recommended in the questionnaire, using the proportion of time that bowel movements looked abnormal according to the Bristol stool form scale. Finally, we collected data on the presence of co‐existing functional dyspepsia (FD), either epigastric pain syndrome (EPS) or postprandial distress syndrome (PDS), according to the Rome IV criteria [27]. Validated questionnaires, detailed below, were used to gather data on physical and psychological health, QoL, and work and activity impairment. Additional information on the scoring metrics for these validated instruments can be found in Table S1.

#### 2.2.2 Assessment of Opioid Use

Participants were asked the following question (verbatim): “Do you take any of the following medications regularly, used for pain, called opiates? (Please tick one or more options)”. Participants were able to select one or more options from: codeine, dihydrocodeine, oxycodone, tramadol, oromorph, morphine, fentanyl, buprenorphine, or none of the above. Those who reported using at least one opioid regularly—both prescribed and over‐the‐counter—were categorised as opioid users.

#### 2.2.3 Gastrointestinal Symptoms and IBS Symptom Severity

All participants were asked to choose their most troublesome symptom from a list of five possibilities: abdominal pain, constipation, diarrhoea, bloating, or urgency. We assessed the severity of symptoms using the IBS severity scoring system (IBS‐SSS) [28], which measures the presence, severity, and frequency of abdominal pain; the presence and severity of abdominal distension; satisfaction with bowel habits; and the degree to which IBS symptoms are affecting, or interfering with, the individual's life.

#### 2.2.4 Psychological and Somatic Symptoms

The hospital anxiety and depression scale (HADS) was used to collect data on anxiety and depression [29]. We collected non‐gastrointestinal somatic symptom data using the patient health questionnaire‐12 (PHQ‐12) [30], derived from the validated PHQ‐15 [31]. Finally, we used the visceral sensitivity index (VSI) [32] to measure the degree of gastrointestinal symptom‐specific anxiety.

#### 2.2.5 Quality of Life: IBS‐Specific and Generic

We used the IBS quality of life (IBS‐QoL), a validated IBS‐specific questionnaire, to measure health‐related QoL in individuals with IBS [33, 34]. To evaluate generic health‐related QoL, we used the EuroQoL [35], which is used widely throughout healthcare research. We used the European QoL 5 dimensions 5 level version (EQ‐5D‐5L) instrument [36], one of the three versions of the EuroQoL. The mean minimal clinically important difference is 0.074 for the EQ‐5D [37] and ranges from 3 to 8 points for the IBS‐QoL [34].

#### 2.2.6 IBS‐Related Resource Use

We collected data on IBS‐related healthcare usage over the 12 months prior to recruitment to the study. We asked participants to report any appointments with healthcare professionals (GPs, gastroenterologists, specialist nurses, dietitians, or psychologists), including the number of appointments, number of investigations (blood tests, stool tests, endoscopies, abdominal ultrasounds, computed tomography scans, magnetic resonance imaging scans, hydrogen breath tests, or 23‐seleno‐25‐homo‐tauro‐cholic acid scans), number of unplanned emergency department attendances or inpatient admissions (including length of stay), and over‐the‐counter and prescribed medication usage (in months). We applied costs for GP appointments from Unit Costs of Health and Social Care 2020 [38], and appointments, investigations, and unplanned inpatient days in secondary care using the National Health Service 2019/20 National Cost Collection Data [39]. We assumed that all the appointments for IBS were follow‐up appointments, which cost less than a new patient appointment. Unit costs for appointments, investigations, and hospital attendances are provided in Table S2. We applied the lowest price for a one‐month supply of each IBS‐related medication using the online version of the British National Formulary [40] (Table S3).

#### 2.2.7 Productivity and Ability to Work in IBS

We used the work productivity and activity impairment questionnaire for IBS (WPAI:IBS) [41]; a validated questionnaire to assess the level of work productivity loss in persons with IBS who are employed, as well as impairment in their activities of daily living. We also used the work and social adjustment scale (WSAS) [42], which has been used by others to measure the effect of IBS on individuals' ability to work, manage at home, engage in social and private leisure activities, and maintain close relationships [43, 44, 45, 46].

#### 2.2.8 Willingness to Accept Risk in Return for Cure of IBS Symptoms

We used a standard gamble to evaluate the risks of death that participants were willing to take in return for an indefinite hypothetical cure of their IBS symptoms [47]. Each question offered participants a choice of a chance of permanent cure of their IBS symptoms with a hypothetical pill or a risk of a painless death in their sleep from the same pill. As the participants moved from one question to the next, the chance of cure was titrated down from 100% while the risk of death was titrated up from 0%. In doing so, we estimated the maximum risk of death that participants were willing to accept for a corresponding minimum chance of cure.

#### 2.2.9 Latent Cluster Analysis

Since IBS is a DGBI, and patients commonly experience anxiety, depression, and somatic symptoms, classification by bowel habit alone perhaps inadequately reflects its multidimensional nature [48]. Latent class analysis identifies unobserved subgroups within a population by incorporating multiple domains simultaneously and generating reproducible, probabilistic classes [49]. This unsupervised approach has challenged the conventional three‐subtype Rome IBS classification system, that is, IBS‐constipation (IBS‐C), IBS‐diarrhoea (IBS‐D), and IBS‐mixed bowel habits (IBS‐M) [50, 51]. Participants in this study were assigned to one of seven clusters using a previously validated latent class analysis model [9, 10, 11, 12] (Table 1). Each patient was placed into one of the seven clusters whose symptom profile best matched their own. Variables used in the original model are provided in Table S4. The prevalence of opioid use was calculated among the participants who belonged to each of the seven clusters and compared between groups.

**TABLE 1:** Symptom profiles of the 7 previously validated clusters [9, 10, 11, 12].

Cluster number	Sample size (*n* = 752)	Cluster description
1	140	Diarrhoea and urgency with low psychological burden
2	195	Low overall gastrointestinal symptom severity with high psychological burden
3	143	Low overall gastrointestinal symptom severity with low psychological burden
4	147	Diarrhoea, abdominal pain, and urgency with high psychological burden
5	24	Constipation, abdominal pain, and bloating with high psychological burden
6	56	High overall gastrointestinal symptom severity with high psychological burden
7	47	Constipation and bloating with low psychological burden

### 2.3 Statistical Analysis

Participants who met the Rome IV criteria for IBS were included in the analysis. We compared the characteristics of individuals who regularly used opioids with those who did not. Categorical variables were analysed using a Chi‐squared test whilst continuous data were analysed using an independent samples t‐test or Mann–Whitney *U* test. Statistical significance was defined as a *p* < 0.01 because of multiple comparisons. A logistic regression model (odds ratio [OR] with 95% confidence interval [CI]), controlling for baseline data that were statistically significant in the univariate analyses, was used to determine the variables that were independently associated with opioid use. To achieve the secondary aim of this study, logistic regression was performed with opioid use as the dependent variable and cluster membership as the independent categorical variable, with Cluster 3 (the lowest‐use group) set as the reference. Analyses were performed using SPSS for Windows (version 27.0 SPSS, Chicago, IL).

## 3 Results

In total, 1278 (29.9%) of the 4280 registrants completed the questionnaire. Among these participants, 752 (58.8%) met the Rome IV criteria for IBS (mean age: 45.3 years [range 18–81 years]; 655 [87.1%] female). Among the 752 individuals with Rome IV IBS, 148 (19.7%) reported using an opioid regularly (Table 2). A greater proportion of opioid users, compared with non‐users, consumed tobacco (*p* = 0.004) and alcohol (*p* < 0.001). A greater proportion of non‐users had a university or postgraduate level of education (*p* < 0.001) and had an annual income of at least £30,000 or more (*p* = 0.001) (Table 2).

**TABLE 2:** Demographics, social history, and gastrointestinal‐related characteristics of IBS.

Variable	Opioid users (*n* = 148)	Non‐users (*n* = 604)	*p* ^a^
Demographics			
Mean age, years (SD)	47.2 (14.3)	44.9 (14.9)	0.10
Female	132 (89.2)	523 (86.6)	0.40
White ethnicity	144 (97.3)	585 (96.9)	0.78
Smoker	26 (17.6)	56 (9.3)	0.004
Alcohol use	60 (40.5)	379 (62.7)	< 0.001
Marital status, married	84 (56.8)	403 (66.7)	0.02
University or postgraduate level of education	39 (26.4)	275 (45.5)	< 0.001
Annual income of £30,000 or more	24 (17.8)	173 (31.7)	0.001
Current employment	71 (48.0)	413 (68.4)	< 0.001
Gastrointestinal‐Related Characteristics of IBS			
IBS Subtype			
IBS‐C	26 (17.6)	110 (18.5)	0.10
IBS‐D	51 (34.5)	255 (42.9)	
IBS‐M	71 (48.0)	230 (38.7)	
Duration of IBS diagnosis, year(s)			
1	4 (2.7)	21 (3.5)	0.71
2	9 (6.1)	32 (5.3)	
3	9 (6.1)	45 (7.5)	
4	4 (2.7)	29 (4.8)	
5	10 (6.8)	28 (4.6)	
> 5	112 (75.7)	449 (74.3)	
Number of IBS‐related drugs in the preceding 12 months			
0	7 (4.7)	89 (14.7)	< 0.001
1	28 (18.9)	161 (26.7)	
2	39 (26.4)	157 (26.0)	
3	26 (17.6)	103 (17.1)	
4	22 (14.9)	54 (8.9)	
≥ 5	26 (17.6)	40 (6.6)	
IBS after acute enteric infection	18 (12.2)	73 (12.1)	0.98
Most troublesome symptom			
Abdominal pain	45 (30.4)	124 (20.5)	0.005
Constipation	11 (7.4)	42 (7.0)	
Diarrhoea	29 (19.6)	88 (14.6)	
Bloating/distension	27 (18.2)	191 (31.6)	
Urgency	36 (24.3)	159 (26.3)	
Rome IV FD			
EPS	60 (40.5)	174 (28.9)	0.006
PDS	79 (53.7)	252 (42.0)	0.01
IBS‐SSS			
Mild	12 (8.1)	74 (12.4)	< 0.001
Moderate	39 (26.4)	261 (43.7)	
Severe	97 (65.5)	262 (43.9)	
Baseline total IBS‐SSS, mean (SD)	323.5 (93.5)	285.6 (94.1)	< 0.001
Baseline IBS‐SSS pain subscore, mean (SD)	114.2 (50.9)	94.1 (54.5)	< 0.001

*Note:* Values are *n* (%) unless stated otherwise.

Abbreviations: EPS, epigastric pain syndrome; FD, functional dyspepsia; IBS, irritable bowel syndrome; IBS‐C, irritable bowel syndrome with constipation; IBS‐D, irritable bowel syndrome with diarrhoea; IBS‐M, irritable bowel syndrome with mixed bowel habits; IBS‐SSS, irritable bowel syndrome symptom severity system; PDS, postprandial distress syndrome.

^a^ Pearson *χ* ^2^ for comparison of categorical data, independent samples *t*‐test for continuous data, and Mann Whitney *U* test for comparison of medians.

**FIGURE 1:**
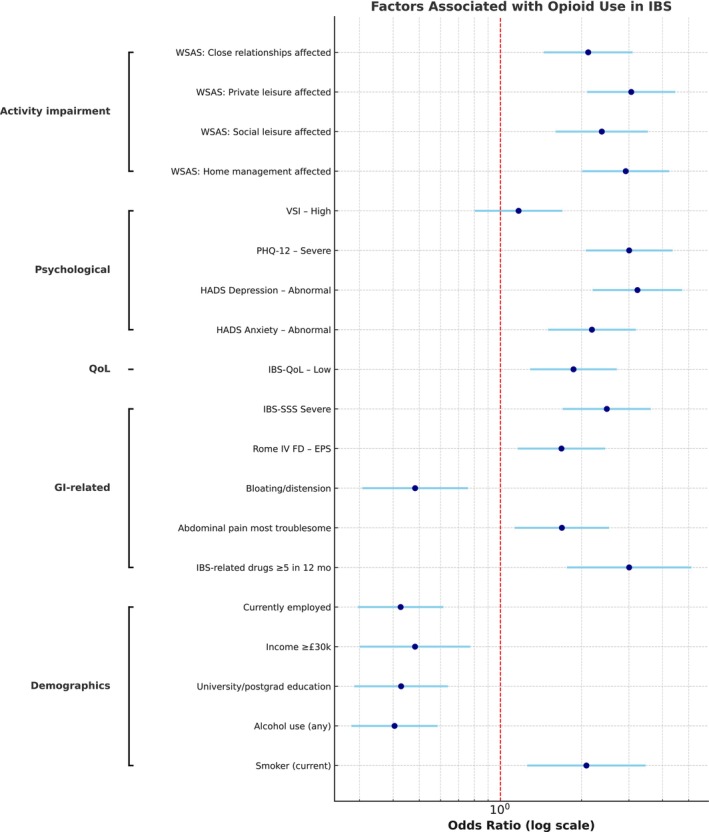
A forest plot displaying the categorical variables most strongly associated with opioid use, restricted to those with statistical significance at *p* < 0.01. Abbreviations: GI, gastrointestinal; HADS, hospital anxiety and depression scale; IBS, irritable bowel syndrome; IBS‐SSS, irritable bowel syndrome severity scoring system; PHQ‐12, patient health questionnaire‐12; QoL, quality of life; Rome IV FD‐EPS, Rome IV functional dyspepsia‐epigastric pain syndrome; VSI, visceral sensitivity index; WSAS, work and social adjustment scale.

Among the 752 participants, 306 (40.7%) had IBS‐D, 301 (40.0%) IBS‐M, 136 (18.1%) IBS‐C, and 9 (1.2%) IBS‐unclassified (IBS‐U). There was no significant difference in the distribution of IBS subtypes among opioid users versus non‐users (*p* = 0.10), nor in the duration of symptoms prior to diagnosis (*p* = 0.71) (Table 2). Four hundred and eleven (54.7%) individuals with IBS met the criteria for Rome IV FD; the EPS subtype of Rome IV FD was more common in people who used opioids (*p* = 0.006). Opioid users had a significantly higher overall IBS‐SSS score versus non‐users (323.5 [93.5] vs. 285.6 [94.1], *p* < 0.001), as well as a higher rating on the IBS‐SSS pain subscale (114.2 [50.9] vs. 94.1 [54.5], *p* < 0.001). Categorical variables most strongly associated with opioid use, restricted to those with statistical significance at *p* < 0.01, are displayed in Figure 1.

The mean IBS‐QoL (41.1 [21.7] vs. 50.1 [22.1], *p* < 0.001) and EQ‐5D‐5L scores (0.346 [0.329] vs. 0.625 [0.241], *p* < 0.001) were significantly lower, indicating worse disease‐specific and generic health‐related QoL, respectively, among those who used opioids (Table 3). There was a significantly higher proportion of individuals with abnormal HADS anxiety or depression scores (*p* < 0.001 for trend for both) among opioid users compared with non‐opioid users. Similarly, a greater proportion of opioid users had severe levels of non‐gastrointestinal somatic symptom reporting on the PHQ‐12 versus non‐users (*p* < 0.001 for trend). There was no difference in the distribution of VSI categories between the two groups (*p* = 0.07). Opioid users were also willing to accept a significantly higher risk of death (4.5 [0.0–10.0] vs. 2.0 [0.0–7.0], *p* = 0.002) in return for a cure of their IBS symptoms from a hypothetical medication.

**TABLE 3:** QoL, psychological symptoms (depression and anxiety), activity impairment, and direct healthcare costs of living with IBS.

Variable	Opioid users (*n* = 148)	Non‐users (*n* = 604)	*p* ^a^
QoL			
IBS‐QoL			
Low	64 (43.2)	175 (29.0)	< 0.001
Medium	53 (35.8)	199 (32.9)	
High	31 (20.9)	230 (38.1)	
IBS‐QoL, mean (SD)	41.1 (21.7)	50.1 (22.1)	< 0.001
EQ‐5D‐5L index, mean (SD)	0.346 (0.329)	0.625 (0.241)	< 0.001
Psychological Symptoms			
HADS Anxiety Categories			
Normal	31 (20.9)	169 (28.0)	< 0.001
Borderline abnormal	20 (13.5)	154 (25.5)	
Abnormal	97 (65.5)	281 (46.5)	
HADS Depression Categories			
Normal	51 (34.5)	353 (58.4)	< 0.001
Borderline abnormal	32 (21.6)	133 (22.0)	
Abnormal	65 (43.9)	118 (19.5)	
PHQ‐12 severity			
Low	2 (1.4)	34 (5.6)	< 0.001
Mild	18 (12.2)	158 (26.2)	
Moderate	52 (35.1)	255 (42.2)	
Severe	76 (51.4)	157 (26.0)	
VSI			
Low	37 (25.0)	210 (34.8)	0.07
Medium	56 (37.8)	191 (31.6)	
High	55 (37.2)	203 (33.6)	
Willingness to accept risk of death in return for cure of IBS symptoms from a hypothetical medication, Median (IQR)	4.5 (0.0–10.0)	2.0 (0.0–7.0)	0.002
Activity Impairment			
WPAI:IBS, median, IQR (higher scores = greater impairment)			
Absenteeism^b^	0.0 (0.0–7.5)	0.0 (0.0–2.6)	0.04
Presenteeism^c^	50.0 (20.0–65.0)	30.0 (10.0–60.0)	0.06
Overall work impairment^d^	34.1 (7.4–61.5)	30.0 (10.0–60.0)	0.88
Activity impairment^e^	60.0 (30.0–80.0)	40.0 (20.0–70.0)	< 0.001
WSAS			
IBS affected home management	72 (48.6)	148 (24.5)	< 0.001
IBS affected social leisure activities	107 (72.3)	316 (52.3)	< 0.001
IBS affected private leisure activities	70 (47.3)	137 (22.7)	< 0.001
IBS affected close relationships	59 (39.9)	144 (23.8)	< 0.001
Mean direct healthcare costs of IBS during the 12 months preceding study recruitment, £, mean (SD)			
Appointments	332.27 (616.09)	198.07 (562.28)	0.01
Investigations	234.30 (456.74)	138.92 (319.3)	0.003
IBS‐related drugs	99.06 (102.3)	66.14 (93.71)	< 0.001
Unplanned attendances	242.26 (705.97)	67.45 (328.65)	< 0.001
Total direct healthcare costs	907.90 (1391.88)	470.58 (892.05)	< 0.001

*Note:* Values are *n* (%) unless stated otherwise.

Abbreviations: EQ‐5D‐5L, European quality of life 5 dimensions 5 level version; HADS, hospital anxiety and depression scale; IBS, irritable bowel syndrome; PHQ‐12, patient health questionnaire‐12; QoL, quality of life; VSI, visceral sensitivity index; WPAI: IBS, work productivity and activity impairment for irritable bowel syndrome; WSAS, work and social adjustment scale.

^a^ Pearson *χ* ^2^ for comparison of categorical data, independent samples *t*‐test for continuous data, and Mann Whitney *U* test for comparison of medians.

^b^ Number of hours missed from work due to health issues: only employed persons answered this question.

^c^ Reduced productivity whilst at work: only employed persons answered this question.

^d^ A combination of absenteeism and presenteeism: only employed persons answered this question.

^e^ The extent to which health problems affect daily activities: score calculated irrespective of employment status.

A greater proportion of non‐users were in active employment compared with opioid users (68.4% vs. 48.0%, *p* = 0.001). Among those employed, there was no significant difference in levels of absenteeism, presenteeism, or overall work impairment scores between opioid users versus non‐users. Opioid users had higher WPAI:IBS activity impairment scores, indicating greater limitations, than non‐users (60.0 [30.0–80.0] vs. 40.0 [20.0–70.0], *p* < 0.001). A greater proportion of opioid users reported that IBS affected their home management, social leisure activities, private leisure activities, and close relationships compared with non‐users (*p* < 0.001 for all analyses).

Mean total direct healthcare costs during the 12 months preceding study recruitment were significantly higher among individuals who used opioids versus non‐users (£907.90 [£1391.90] vs. £470.58 [£892.10], *p* < 0.001), which reflected higher mean costs of investigations (*p* = 0.003), IBS‐related drugs (*p* < 0.001), and unplanned hospital attendances (*p* < 0.001) in the opioid group (Table 3).

### 3.1 Latent Class Analysis

There was a statistically significant difference in the distribution of opioid use among the seven clusters (Table 4), *p* < 0.001. The prevalence of opioid use was lowest in Cluster 3 (7.0%) and highest in Cluster 6 (46.4%). Using Cluster 3 as the comparator, logistic regression confirmed that patients in Cluster 6 were significantly more likely to report opioid use (OR 11.53, 95% CI 5.03–26.43, *p* < 0.001). Intermediate rates of opioid consumption were reported in Cluster 1 (17.1%), Cluster 2 (21.5%), Cluster 4 (23.1%), Cluster 5 (25.0%), and Cluster 7 (12.8%).

**TABLE 4:** The distribution of opioid use among the seven clusters.

Cluster number	Sample size, *n*	Opioid users, *n* (%)	OR (95% CI) vs. C3	*p*
C3 (reference)	143	10 (7.0)	—	—
C1	140	24 (17.1)	2.75 (1.26–5.99)	0.01
C2	195	42 (21.5)	3.65 (1.76–7.56)	< 0.001
C4	147	34 (23.1)	4.00 (1.89–8.46)	< 0.001
C5	24	6 (25.0)	4.43 (1.44–13.66)	0.01
C6	56	26 (46.4)	11.53 (5.03–26.43)	< 0.001
C7	47	6 (12.8)	1.95 (0.67–5.68)	0.22

### 3.2 Multivariate Logistic Regression Model to Identify Baseline Characteristics Associated With Opioid Consumption

In the multivariate logistic regression model controlling for data that were statistically significant in the univariate analyses (Table S5), lower generic health‐related QoL (worse EQ‐5D‐5L) was associated with opioid use (0.088 [0.028–0.280], *p* < 0.001). Overall, the regression model was statistically significant (Omnibus *χ* ^2^ = 150.6, *p* < 0.001), explained 31.9% of the variance in opioid use (Nagelkerke *R* ^2^), and showed acceptable calibration (Hosmer–Lemeshow *p* = 0.07).

## 4 Discussion

In this large cross‐sectional study of individuals with Rome IV IBS, approximately one in five people used opioids regularly. A greater proportion of opioid users reported abdominal pain, met the diagnostic criteria for Rome IV EPS subtype of FD, and had higher IBS symptom severity scores compared with non‐users. In addition, opioid users had higher levels of anxiety, depression, gastrointestinal symptom‐specific anxiety, somatoform‐symptom reporting, and poorer disease‐specific and generic health‐related QoL compared with non‐users. Compared with non‐users, opioid users had greater activity impairment and higher healthcare costs over the preceding 12 months, including those related to unplanned hospital attendances, investigations, and IBS‐related drugs. A multivariate regression model demonstrated an association between generic health‐related QoL (worse EQ‐5D‐5L) and opioid use.

The prevalence of opioid use in this study is comparable to that seen in a North American community‐based study of IBS [52], but lower than the prevalence in a tertiary care population of patients with IBS‐C [18]. Compared with non‐users, opioid users had higher levels of anxiety and depression, consistent with data from the Veterans Health Administration registry among people with various DGBI [53] and with findings among patients with inflammatory bowel disease [54, 55]. It remains unclear whether these elevated scores act as confounders contributing to opioid use or if they are consequences resulting from opioid use itself.

Opioid users in our study expressed a greater willingness to accept the risk of death in return for a cure of IBS symptoms from a hypothetical medication. This observation may be explained, at least in part, by potential confounders: opioid users had greater IBS symptom severity, were more likely to take additional medications for IBS in the 12 months prior to enrolment, and had more abnormal HADS depression scores—each previously associated with the risk of death in a previous study of individuals with IBS [20]. These data add to existing evidence demonstrating that 14% of patients would risk a 1/1000 risk of death in order to receive a treatment that would make them symptom‐free [52].

Although opioid prescribing was not included as a clustering variable, its distribution mapped closely onto the expected clinical profiles—being lowest in cluster 3 (relatively mild gastrointestinal and psychological symptoms) and highest in cluster 6 (severe IBS symptoms, high psychiatric comorbidity, and marked functional impairment). This external association strengthens the construct validity of the subgroups and highlights their potential clinical utility. These findings suggest that cluster‐based approaches could help identify patients at highest risk of inappropriate opioid prescribing.

The proportion of participants with severe non‐gastrointestinal somatic symptoms, determined using the PHQ‐12, was approximately twice as high among opioid users versus non‐users. These findings align with evidence demonstrating an association between opioid intake and clinically significant fatigue in patients with lower back pain [56]. It is conceivable that participants in our study used opioids to manage non‐visceral somatic symptoms, although it is equally possible that opioids triggered and perpetuated somatic symptoms via central sensitisation [6].

The mean EQ‐5D‐5L score in our sample of patients' users was comparable to that reported by individuals with opioid‐induced constipation in Norway [57]. However, the mean EQ‐5D‐5L QoL score was lower among opioid users in our study compared with non‐users, and this difference met the minimal clinically important difference threshold. Importantly, the multivariate logistic regression model demonstrated an association between opioid intake and generic health‐related QoL (worse EQ‐5D‐5L). These findings are unsurprising given that opioid users in our cohort had worse psychological symptoms and greater somatic symptom reporting, both of which have been shown to be associated with poorer QoL in a previous study [58].

Opioid users reported greater activity impairment than non‐users, but no significant differences were observed in absenteeism, presenteeism, or overall work impairment. As these three latter domains are assessed only in employed individuals, the lack of statistical difference may be attributed to the higher unemployment rate observed among opioid users in this study. Consistent with the results of this study, patients with gastroparesis who consume opioids for chronic pain have a lower employment rate and work fewer hours per week versus non‐users [59].

Unplanned hospital attendances were greater among opioid users compared with non‐users. This has also been demonstrated in a single‐centre UK tertiary care setting of patients with IBS‐C [18] and FD [60]. Whether this reflects opioid initiation following a hospital admission, as observed in a USA electronic health record database of adults with IBS [61], or the greater frequency of hospital admissions secondary to opioid side‐effects cannot be confirmed in our cross‐sectional study.

A key strength of this study is its large sample size of individuals meeting the Rome IV criteria for IBS, all of whom completed validated questionnaires. We also assessed a broad range of extra‐intestinal outcomes, including psychological symptoms, QoL, impact on work and activities of daily living, and healthcare costs during the 12 months preceding study recruitment.

The cross‐sectional design of this study, which limits causal inference, has several additional limitations. We were unable to check participants' medical records to rule out other organic diseases that present with similar symptoms, such as coeliac disease or inflammatory bowel disease. However, 90% of participants in the registry had seen a GP or gastroenterologist for their IBS, and nearly 80% had a diagnosis of IBS for 5 years or longer. These statistics support a “true” Rome IV IBS in the majority, if not most, participants recruited in this study. Importantly, we recruited a self‐selecting group of individuals who consented to be part of the study, so outcomes may not be generalisable to all people with Rome IV IBS in community settings, nor to minority ethnic groups who were under‐represented in this study population. The 90% healthcare‐seeking rate in this sample of patients is significantly higher than the 30%–50% reported in other UK settings [62], suggesting that the ContactMe population may be more likely to seek care and use medication compared to the “typical” patient with IBS. Since this study was not designed to test causality, and the regression analyses examined predictors rather than outcomes, associations with higher IBS‐SSS and depression cannot determine directionality and may reflect either predictors or consequences of opioid use. Also, indications for opioid use (e.g., post‐operative pain management) and duration of opioid use were not defined; hence, some prescriptions may reflect non‐IBS pain, and chronic use could exacerbate symptoms through narcotic bowel syndrome, potentially influencing outcomes. Nonetheless, our findings support BSG guidelines for managing IBS, which highlight the iatrogenic harms associated with opioid use in IBS [15].

In conclusion, approximately one in five individuals with Rome IV IBS in the UK consume opioids regularly. Compared with non‐users, opioid users had a greater burden of gastrointestinal and psychological symptoms, worse QoL, greater work and activity impairment, and higher healthcare costs. Future studies should evaluate the prognostic significance of both the duration and potency of opioid use.

## Author Contributions

**Mohsin F. Butt:** writing – original draft. **Vivek C. Goodoory:** conceptualization, methodology, formal analysis. **Cho Ee Ng:** data curation, methodology. **Christopher J. Black:** writing – review and editing, formal analysis, conceptualization. **Alexander C. Ford:** conceptualization, writing – review and editing, formal analysis. **Maura Corsetti:** writing – review and editing, supervision. **Peter Paine:** writing – review and editing, supervision, conceptualization.

## Conflicts of Interest

The authors declare no conflicts of interest.

## Supporting information


**Table S1:** Scoring Details for Validated Instruments. EQ‐5D‐5L, European quality of life 5 dimensions 5 level version; HADS, hospital anxiety and depression scale; IBS, irritable bowel syndrome; IBS‐QoL, irritable bowel syndrome quality of life; IBS‐SSS, irritable bowel syndrome symptom severity system; PHQ‐12, patient health questionnaire‐12; QoL, quality of life; VSI, visceral sensitivity index; WPAI:IBS, work productivity and activity impairment questionnaire for irritable bowel syndrome; WSAS, work and social adjustment scale.
**Table S2:** Unit Costs (in UK pounds) for IBS‐related Appointments, Investigations, and Unplanned Hospital Attendances or Admissions. A&E, accident and emergency; CT, computed tomography; GP, general practitioner; MRI, magnetic resonance imaging; SeHCAT, Selenium‐75‐homocholic acid taurine.
**Table S3:** Unit Costs (in UK pounds) for a 1‐month Supply of IBS‐related Medications.
**Table S4:** Variables Used in the Latent Class Analysis. DGBI, disorder of gut‐brain interaction; HADS, hospital anxiety and depression scale; IBS, irritable bowel syndrome; PHQ‐12, patient health questionnaire‐12.
**Table S5:** Variables included in the multivariate logistic regression model. P values < 0.01 were considered statistically significant and only then were complete data shown. EPS, epigastric pain syndrome; EQ‐5D‐5L, EuroQoL 5‐Dimension 5‐Level; FD, functional dyspepsia; HADS, hospital anxiety and depression scale; IBS, irritable bowel syndrome; IBS‐SSS; irritable bowel syndrome symptom severity system; N/S, not [statistically] significant; PHQ, patient health questionnaire; QoL, quality of life; WSAS, work and social adjustment scale.

## Data Availability

The data that support the findings of this study are available from the corresponding author upon reasonable request.
