# Supplementary material for: Prevalence of Opioid Use and Associated Healthcare Outcomes in Rome IV Irritable Bowel Syndrome in the United Kingdom
Source: Aliment Pharmacol Ther. 2025 Oct 8;63(4):557–68. doi: 10.1111/apt.70400 (PMC12854644; doi:10.1111/apt.70400)
Supplement: Supplementary file 1 — Table S1: Scoring Details for Validated Instruments. EQ‐5D‐5L, European quality of life 5 dimensions 5 level version; HADS, hospital anxiety and depression scale; IBS, irritable bowel syndrome; IBS‐QoL, irritable bowel syndrome quality of life; IBS‐SSS, irritable bowel syndrome symptom severity system; PHQ‐12, patient health questionnaire‐12; QoL, quality of life; VSI, visceral sensitivity index; WPAI:IBS, work productivity and activity impairment questionnaire for irritable bowel syndrome; WSAS, work and social adjustment scale. Table S2: Unit Costs (in UK pounds) for IBS‐related Appointments, Investigations, and Unplanned Hospital Attendances or Admissions. A&E, accident and emergency; CT, computed tomography; GP, general practitioner; MRI, magnetic resonance imaging; SeHCAT, Selenium‐75‐homocholic acid taurine. Table S3: Unit Costs (in UK pounds) for a 1‐month Supply of IBS‐related Medications. Table S4: Variables Used in the Latent Class Analysis. DGBI, disorder of gut‐brain interaction; HADS, hospital anxiety and depression scale; IBS, irritable bowel syndrome; PHQ‐12, patient health questionnaire‐12. Table S5: Variables included in the multivariate logistic regression model. P values < 0.01 were considered statistically significant and only then were complete data shown. EPS, epigastric pain syndrome; EQ‐5D‐5L, EuroQoL 5‐Dimension 5‐Level; FD, functional dyspepsia; HADS, hospital anxiety and depression scale; IBS, irritable bowel syndrome; IBS‐SSS; irritable bowel syndrome symptom severity system; N/S, not [statistically] significant; PHQ, patient health questionnaire; QoL, quality of life; WSAS, work and social adjustment scale. [file APT-63-557-s001.docx]

**Supplementary Table 1.** Scoring Details for Validated Instruments. Abbreviations: EQ-5D-5L, European quality of life 5 dimensions 5 level version; HADS, hospital anxiety and depression scale; IBS, irritable bowel syndrome; IBS-QoL, irritable bowel syndrome quality of life; IBS-SSS, irritable bowel syndrome symptom severity scale; PHQ-12, patient health questionnaire-12; QoL, quality of life; VSI, visceral sensitivity index; WSAS, work and social adjustment scale; WPAI:IBS, work productivity and activity impairment questionnaire for irritable bowel syndrome

| **Instrument** | **Details on Scoring** |
| --- | --- |
| IBS-SSS | The IBS-SSS carries a maximum score of 500 points, with <75 points indicating remission of symptoms; 75-174 points mild symptoms; 175-299 points moderate symptoms; and 300-500 points severe symptoms. |
| HADS | The total HADS score ranges from 0 to 21 for either anxiety or depression. We categorized severity for each into normal (total HADS depression or anxiety score 0-7), borderline normal (8-10), or abnormal (≥11). |
| PHQ-12 | The total PHQ-12 score ranges from 0 to 24. We categorized severity into high (total PHQ-12 ≥13), medium (8-12), low (4-7), or minimal (≤3). |
| VSI | Responses to each of the 15 items are provided on a six-point scale from “strongly disagree” (score 0) to “strongly agree” (score 5). We divided these data into equally sized tertiles, as there are no validated cut offs to define low, medium, or high levels of gastrointestinal symptom-specific anxiety. |
| IBS-QoL | The IBS-QoL consists of 34 items, each ranked on a five-point Likert scale ranging from 0 to 4, with a total possible score of 0-136 and lower scores indicating better QoL. The 34 items are based on the following eight variables: dysphoria, interference with activity, body image, health worry, food avoidance, social reactions, sexual activity, and relationships. Score were transformed to a 0 to 100-point scale with zero indicating the worst QoL and 100 indicating best QoL. We divided these data into equally sized tertiles, as there are no validated cut offs to define low, medium, or high levels of QoL. |
| EQ-5D-5L | The EQ-5D-5L consists of five items covering different aspects of health: mobility, self-care, ability to carry out usual activities, pain/discomfort, and anxiety/depression. Each item has five levels of responses, allowing for a total of 3125 possible health states. We mapped each health state to obtain a utility score for a UK population using a crosswalk calculator(54). |
| WPAI:IBS | The WPAI:IBS consists of six questions related to current employment status, hours of work missed due to IBS, hours of work missed due to other reasons, hours actually worked, the degree to which IBS has affected work productivity whilst working, and the degree to which IBS has affected other activities of daily living in the last seven days. The WPAI:IBS measures four domains: absenteeism, which is the percentage of work hours missed because of IBS; presenteeism, which is the percentage of impairment experienced whilst working because of IBS; overall work impairment, which is the percentage of work productivity loss; and activity impairment, which is the percentage impairment in activities of daily living. |
| WSAS | The five domains are scored on a 9-point scale from “not at all” (score 0) to “very severely” (score 8). |

**Supplementary Table 2.** Unit Costs (in UK pounds) for IBS-related Appointments, Investigations, and Unplanned Hospital Attendances or Admissions. Abbreviations: A&E, accident and emergency; CT, computed tomography; GP, general practitioner; MRI, magnetic resonance imaging; SeHCAT, Selenium-75-homocholic acid taurine

| **Item** | **Cost (£)** |
| --- | --- |
| Follow-up appointment with a GP | 33.00 |
| Follow-up appointment with a gastroenterologist | 148.12 |
| Follow-up appointment with a specialist nurse | 127.91 |
| Follow-up appointment with a dietitian | 83.03 |
| Follow-up appointment with a psychologist | 179.84 |
| Blood test | 1.81 |
| Stool test | 8.09 |
| Gastroscopy | 482.23 |
| Colonoscopy | 559.35 |
| Hydrogen breath test | 57.96 |
| Abdominal ultrasound | 62.39 |
| Abdominal and pelvis CT scan | 114.36 |
| Abdominal MRI scan | 144.29 |
| SeHCAT scan | 367.73 |
| A&E attendance | 220.53 |
| Inpatient admission under gastroenterology | 1551.77 |

**Supplementary Table 3.** Unit Costs (in UK pounds) for a 1-month Supply of IBS-related Medications.

| **Medication** | **Cost (£)** |
| --- | --- |
| Loperamide | 1.68 |
| Sodium picosulfate | 4.62 |
| Bisacodyl | 1.67 |
| Polyethylene glycol | 2.99 |
| Hyoscine | 9.63 |
| Alverine | 7.64 |
| Mebeverine | 4.39 |
| Dicycloverine | 30.00 |
| Ispaghula | 3.24 |
| Peppermint oil | 4.95 |
| Amitriptyline | 1.08 |
| Nortriptyline | 1.00 |
| Imipramine | 2.15 |
| Fluoxetine | 0.50 |
| Paroxetine | 1.26 |
| Sertraline | 0.80 |
| Citalopram | 1.02 |
| Escitalopram | 1.55 |
| Lubiprostone | 53.48 |
| Linaclotide | 37.56 |
| Prucalopride | 47.62 |
| Eluxadoline | 88.20 |

**Supplementary Table 4.** Variables Used in the Latent Class Analysis. Abbreviations: DGBI, disorder of gut-brain interaction; HADS, Hospital Anxiety and Depression Scale; IBS, irritable bowel syndrome; PHQ-12, patient health questionnaire-12.

|  | **Variable** | **Type of variable** | **Measurement scale** | **Reason for including in the model** |
| --- | --- | --- | --- | --- |
| Gastrointestinal symptoms | Frequency of abdominal pain anywhere in the abdomen in the past 3 months | Ordinal | 9-point scale from never (score, 0) to multiple times per day or all the time (score, 8) | All of these variables for quantifying gastrointestinal symptoms were taken from Rome Foundation questionnaires These are the recognized gold standard for diagnosing IBS and are used widely |
|  | Frequency of abdominal pain being closely related to a bowel movement | Ordinal | 11-point scale from 0% (never) to 100% (always) |  |
|  | Frequency with which abdominal pain improved or resolved after a bowel movement | Ordinal | 11-point scale from 0% (never) to 100% (always) |  |
|  | Frequency with which stools became softer or harder than usual in association with abdominal pain | Ordinal | 11-point scale from 0% (never) to 100% (always) |  |
|  | Frequency with which stools became more or less frequent than usual in association with abdominal pain | Ordinal | 11-point scale from 0% (never) to 100% (always) |  |
|  | Frequency with which abdominal pain started or got worse after a meal | Ordinal | 11-point scale from 0% (never) to 100% (always) |  |
|  | Frequency with which abdominal pain restricted usual activities | Ordinal | 11-point scale from 0% (never) to 100% (always) |  |
|  | Frequency of hard or lumpy stools in the past 3 months | Ordinal | 5-point scale from 0% (never or rarely) to 100% (always) |  |
|  | Frequency of loose, mushy, or watery stools in the past 3 months | Ordinal | 5-point scale from 0% (never o rarely) to 100% (always) |  |
|  | Frequency of faecal urgency over the past 3 months | Ordinal | 9-point scale from never (score, 0) to multiple times per day or all the time (score, 8) |  |
|  | Frequency of faecal incontinence over the past 3 months | Ordinal | 9-point scale from never (score, 0) to multiple times per day or all the time (score, 8) |  |
|  | Frequency of abdominal bloating or distension over the past 3 months | Ordinal | 9-point scale from never (score, 0) to multiple times per day or all the time (score, 8) |  |
| Extraintestinal symptoms | All individual items of the PHQ-12 and the frequency experienced in the past 4 weeks: Back pain Arm, leg, joint pain Period pain/period problems Headaches Chest pain Dizziness Fainting spells Heart pounding/racing Shortness of breath Pain/problems during sex Feeling tired or low in energy Trouble sleeping | Ordinal | 3-point scale: never (score, 0), a little (score, 1), or a lot (score, 2) | Reporting symptoms referable to multiple body systems, also referred to as *somatization*, is recognized as being associated with IBS and other DGBI.  The PHQ-12 questionnaire is a widely used and validated method for measuring this |
| Mood | Presence of anxiety, as measured by the total score of the HADS-Anxiety questionnaire | Ordinal | 3-point scale: normal (score, 0), borderline (score, 1), or abnormal (score, 2) | Abnormal mood is well recognized as being an important factor in IBS The HADS questionnaire for quantifying the presence of anxiety and/or depression are used widely and validated for this purpose |
|  | Presence of depression, as measured by the total score of HADS-Depression questionnaire | Ordinal | 3-point scale: normal (0), borderline (1), or abnormal (2) |  |

**Supplementary Table 5.** Variables included in the multivariate logistic regression model. P values <0.01 were considered statistically significant and only then were complete data shown. Abbreviations: EPS, epigastric pain syndrome; EQ-5D-5L, EuroQoL 5-Dimension 5-Level; FD, functional dyspepsia; HADS, hospital anxiety and depression scale; IBS, irritable bowel syndrome; IBS-SSS; irritable bowel syndrome symptom severity scale; N/S, not [statistically] significant; PHQ, patient health questionnaire; QoL, quality of life; WSAS, work and social adjustment scale.

| **Variables** | **OR (95% CI)** | **P value** |
| --- | --- | --- |
| EQ-5D-5L index | 0.088 (0.028-0.280) | <0.001 |
| Cigarette smoking (1) | N/S | N/S |
| Alcohol intake (1) | N/S | N/S |
| Most Troublesome Symptom (1) | N/S | N/S |
| Most Troublesome Symptom (2) | N/S | N/S |
| Most Troublesome Symptom (3) | N/S | N/S |
| Most Troublesome Symptom (4) | N/S | N/S |
| HADS anxiety category at baseline (1) | N/S | N/S |
| HADS anxiety category at baseline (2) | N/S | N/S |
| HADS depression category at baseline (1) | N/S | N/S |
| HADS depression category at baseline (2) | N/S | N/S |
| IBS-SSS severity at baseline (1) | N/S | N/S |
| IBS-SSS severity at baseline (2) | N/S | N/S |
| PHQ-12 severity at baseline (1) | N/S | N/S |
| PHQ-12 severity at baseline (2) | N/S | N/S |
| PHQ-12 severity at baseline (3) | N/S | N/S |
| Income ≥30,000 GBP per year (1) | N/S | N/S |
| Number of drugs prescribed in the last 12 months categories (1) | N/S | N/S |
| Number of drugs prescribed in the last 12 months categories (2) | N/S | N/S |
| Number of drugs prescribed in the last 12 months categories (3) | N/S | N/S |
| Number of drugs prescribed in the last 12 months categories (4) | N/S | N/S |
| Number of drugs prescribed in the last 12 months categories (5) | N/S | N/S |
| University or postgraduate level of education (1) | N/S | N/S |
| WSAS, IBS affected home management (1) | N/S | N/S |
| WSAS, IBS affected social leisure activities (1) | N/S | N/S |
| WSAS, IBS affected private leisure activities (1) | N/S | N/S |
| WSAS, IBS affected close relationships (1) | N/S | N/S |
| IBS-QoL (0-100) | N/S | N/S |
| FD – EPS (1) | N/S | N/S |
| Rome IV baseline cluster assignment (1) | N/S | N/S |
| Rome IV baseline cluster assignment (2) | N/S | N/S |
| Rome IV baseline cluster assignment (3) | N/S | N/S |
| Rome IV baseline cluster assignment (4) | N/S | N/S |
| Rome IV baseline cluster assignment (5) | N/S | N/S |
| Rome IV baseline cluster assignment (6) | N/S | N/S |
| Active employment (1) | N/S | N/S |
| Standard gamble death (1) | N/S | N/S |
| Cost of all investigations | N/S | N/S |
| Cost of all drugs | N/S | N/S |
| Cost of unplanned unattendances | N/S | N/S |
| Total costs | N/S | N/S |
